# Circulating Total Cell-Free DNA Levels Are Increased in Hypertensive Disorders of Pregnancy and Associated with Prohypertensive Factors and Adverse Clinical Outcomes

**DOI:** 10.3390/ijms22020564

**Published:** 2021-01-08

**Authors:** Lorena M. Amaral, Valeria C. Sandrim, Matthew E. Kutcher, Frank T. Spradley, Ricardo C. Cavalli, Jose E. Tanus-Santos, Ana C. Palei

**Affiliations:** 1 Department of Pharmacology and Toxicology, School of Medicine, University of Mississippi Medical Center, Jackson, MS 39216-4500, USA; lmamaral@umc.edu; 2 Department of Biophysics and Pharmacology, Institute of Biosciences, Universidade Estadual Paulista, Botucatu, SP 18618-689, Brazil; valeria.sandrim@unesp.br; 3 Department of Surgery, School of Medicine, University of Mississippi Medical Center, Jackson, MS 39216-4500, USA; mkutcher@umc.edu (M.E.K.); fspradley@umc.edu (F.T.S.); 4 Department of Gynecology and Obstetrics, Ribeirao Preto Medical School, University of Sao Paulo, Ribeirao Preto, SP 14049-900, Brazil; rcavalli@fmrp.usp.br; 5 Department of Pharmacology, Ribeirao Preto Medical School, University of Sao Paulo, Ribeirao Preto, SP 14049-900, Brazil; tanus@fmrp.usp.br

**Keywords:** adverse maternal-fetal outcomes, biomarkers, cell-free DNA, gestational hypertension, preeclampsia

## Abstract

Previous studies have described increased circulating cell-free DNA (cfDNA) in hypertensive disorders of pregnancy (HDP). Here, we aimed first to confirm this information using a simple, but sensible fluorescent assay, and second to investigate whether total cfDNA is associated with circulating factors known to be linked to the pathophysiology of HDP as well as with poor maternal-fetal outcomes. We studied 98 women with healthy pregnancies (HP), 88 with gestational hypertension (GH), and 91 with preeclampsia (PE). Total DNA was extracted from plasma using the QIAamp DNA blood mini kit and quantified using Quant-iT™ PicoGreen^®^ dsDNA fluorescent detection kit. We found higher total cfDNA levels in GH and PE (197.0 and 174.2 ng/mL, respectively) than in HP (140.5 ng/mL; both *p* < 0.0001). Interestingly, total cfDNA levels were elevated in both male and female-bearing pregnancies diagnosed with either HDP, and in more severe versus less severe HDP cases, as classified according to responsiveness to antihypertensive therapy. In addition, total cfDNA was independently associated with HDP, and a cutoff concentration of 160 ng/mL provided appropriate sensitivity and specificity values for diagnosing GH and PE compared to HP (70–85%, both *p* < 0.0001). Moreover, high total cfDNA was associated with adverse clinical outcomes (high blood pressure, low platelet count, preterm delivery, fetal growth restriction) and high prohypertensive factors (sFLT-1, sEndoglin, MMP-2). These findings represent a step towards to the establishment of cfDNA as a diagnostic tool and the need to understand its role in HDP.

## 1. Introduction

Hypertensive disorders of pregnancy (HDP) constitute a clinically challenging group of pregnancy complications. They affect 5–10% of all pregnancies [1,2] and constitute a leading cause of maternal, fetal, and infant morbidity and mortality worldwide [1,3]. Gestational hypertension (GH) is defined as new-onset hypertension after 20 weeks of gestation; in recognition of its syndromic nature, recent guidelines define preeclampsia (PE) as gestational hypertension accompanied by hematologic, liver, renal, cerebral, and/or visual disturbances [4,5]. Despite the global impact of these disorders, prophylactic and definitive treatments for HDP are lacking, and identifying biomarkers that may predict the development, severity, and/or treatment responsiveness of these potentially life-threatening conditions are of utmost importance [6,7]. The establishment of biomarkers and their association with unfavorable clinical outcomes may shed light on novel therapeutic targets.

Multiple pathways have been associated with the pathophysiology of PE, including inadequate placental development and systemic maternal endothelial dysfunction [8]. Providing an insight into potential mechanisms linking impaired placentation and endothelial function, both maternal and fetal-derived cell-free deoxyribonucleic acid (cfDNA) fragments have been found in the circulation of pregnant women [9,10], and are known to be elevated in HDP compared to normal pregnancy [11,12,13,14,15,16,17,18,19,20,21,22,23]. In addition, increased cfDNA levels in maternal circulation correlate with disease severity in PE, and are associated with poor maternal-fetal outcomes [11,12,13,14,17,20,22,23,24,25,26,27].

The exact underlying mechanism leading to the release of extracellular DNA into the maternal circulation and its consequences are not completely clear, but existing evidence indicates that fetal cell trafficking is perturbed in placental tissue early in gestation, and predates the manifestation of clinical symptoms in PE [28]. It has been hypothesized that increased circulating levels of fetal cfDNA may be due to ischemia/hypoxia within the intervillous space resulting in enhanced oxidative stress, apoptosis and/or necrosis of trophoblastic cells, and resultant shedding of fetal cfDNA into the maternal circulation [29], potentially compounded by reduced clearance of fetal cfDNA in PE [30]. However, uncertainty remains regarding the specific cellular source of circulating maternal cfDNA. Recent studies suggest that fetal cfDNA may activate specific receptors in maternal immune cells and platelets, which in turn trigger the production and secretion of pro-inflammatory cytokines and additional cfDNA release, resulting in a systemic humoral and cellular immune response in PE [31]. Interestingly, cfDNA may also act on the maternal vasculature, possibly contributing to endothelial and vascular dysfunction and then hypertension in PE [32].

Therefore, this study aimed to characterize levels of total cfDNA in healthy pregnancy (HP), GH, and PE via fluorescence spectrometry (a simple, rapid, and sensible benchtop test that could easily be translated to bedside clinical practice). To accomplish this, we performed a prospective observational study of pregnant women carried by our hospital’s Gynecology and Obstetrics group, collecting plasma at a single time point during clinic evaluation for confirmation of HP, GH, or PE diagnosis, and following enrolled participants for maternal and fetal-related outcomes until post-partum. We hypothesized that (i) plasma total cfDNA levels are increased in GH compared to HP and in PE compared to both HP and GH, and (ii) plasma total cfDNA levels are associated with circulating factors known to be disrupted in HDP as well as with disease severity and adverse maternal-fetal outcomes. Moreover, we evaluated clinically relevant cutoff values of plasma total cfDNA that may assist with the diagnosis of HPD.

## 2. Results

Table 1 summarizes the clinical characteristics of the 277 pregnant women included in the present study. There were no significant differences in age, race, smoking status, primiparity, or heart rate (HR); no differences in clinical laboratory values including hematocrit, hemoglobin, white blood cell and neutrophil counts, platelet count, alanine transaminase (ALT), aspartate aminotransferase (AST), and lactate dehydrogenase (LDH); and no difference in placental weight among study groups (all *p* > 0.05). Systolic (SBP) and diastolic (DBP) blood pressure values were higher in GH and PE than in HP (all *p* < 0.05), even though most patients with HDP were on antihypertensive drug therapy: methyldopa (1000–1500 mg per day), nifedipine (40–60 mg per day), and/or hydralazine (5–30 mg). Moreover, PE exhibited greater SBP and DBP values and a higher frequency of non-responsiveness to antihypertensive therapy than GH (all *p* < 0.05). As markers of reduced renal function and increased renal damage, plasma creatinine levels, as well as the degree of proteinuria, were increased in PE compared to GH (both *p* < 0.05). Although creatinine and proteinuria were not assessed in HP, all of these women exhibited normal urine dipstick tests. Plasma glucose levels were increased in GH and PE compared to HP (both *p* < 0.05); however, none of these patients met the criteria for a diagnosis of chronic or gestational diabetes. Furthermore, body mass index (BMI) at enrollment was higher in GH and PE than in HP (both *p* < 0.05). Lower gestational age (GA) at enrollment, GA at delivery, and newborn weight, as well as a higher frequency of preterm delivery, were found in PE compared to both HP and GH (all *p* < 0.05). Additionally, the frequency of fetal growth restriction (FGR) was higher in GH and PE than in HP (both *p* < 0.05). Other maternal complications included 6 PE patients who experienced hemolysis, elevated liver enzymes and low platelet count (HELLP) syndrome, plus 1 GH and 1 PE patient who had imminent eclampsia and, thus, were treated with magnesium sulfate to avoid the development of seizures.

**Figure 1:**
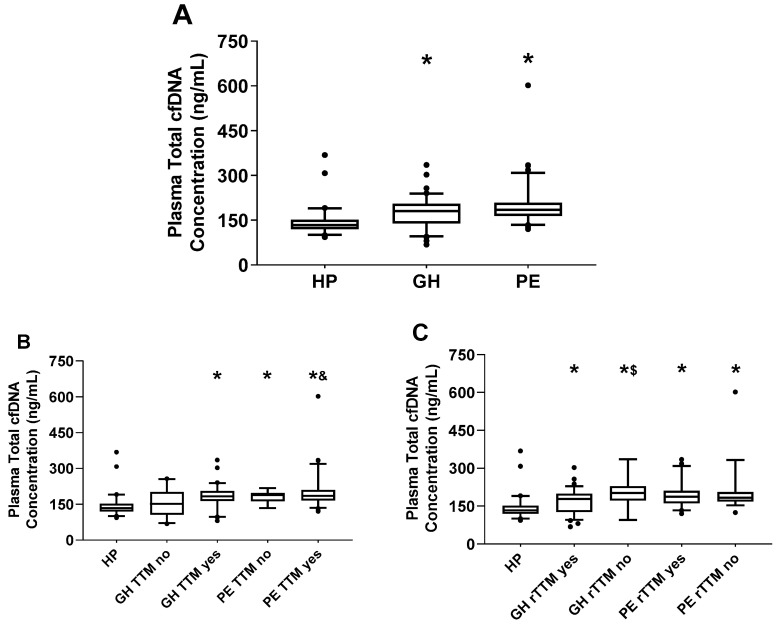
Total cell-free DNA (cfDNA) concentration in plasma of women with healthy pregnancies (HP), gestational hypertension (GH), or preeclampsia (PE) (**A**). Total cfDNA levels in patients prescribed (treatment (TTM) yes) or not prescribed (TTM no) antihypertensive therapy by HDP status, compared to HP (**B**). Total cfDNA levels in women with GH or PE who were treatment-responsive (rTTM yes) versus non-responsive (rTTM no) to antihypertensive therapy, compared to HP (**C**). Box and whiskers are median {interquartile range, 5–95% percentile}. * *p* < 0.0001 versus HP group and ^&^ *p* < 0.05 versus GH TTM no group based on Kruskal-Wallis test with Dunn’s multiple comparisons correction. ^$^ *p* < 0.05 versus GH rTTM yes group based on Mann-Whitney test.

At study enrollment, blood was drawn and plasma samples were stored for batch processing of extraction and quantification of total cfDNA. Based on fluorescence spectrometry, total cfDNA concentrations were higher in plasma from GH and PE than from HP (both *p* < 0.0001; Figure 1A); however, total cfDNA was not different between GH and PE (*p* > 0.05; Figure 1A). In a sub-analysis of these data comparing total cfDNA only in those women with fetal sex reported in medical records (Supplementary Materials Figure S1), there were no differences in male versus female bearing-pregnancies within each study group (all *p* > 0.05), and levels remained increased in both GH and PE compared to HP (all *p* < 0.05).

Next, we determined whether antihypertensive drugs affect plasma total cfDNA and whether these levels vary according to disease severity. In this study, antihypertensive therapy was used as a proxy of disease severity, with the notion that HDP patients under drug treatment and those considered non-responsive to these drugs manifest more severe clinical symptoms. First, when pregnant women with HDP were grouped into those prescribed versus those not prescribed antihypertensive therapy (see Materials and Methods Section for antihypertensive therapy eligibility), total cfDNA was elevated in GH with treatment (TTM yes), PE without treatment (TTM no), and PE with treatment compared to HP (all *p* < 0.0001; Figure 1B); however, cfDNA levels in GH without treatment were similar to HP (*p* > 0.05; Figure 1B), and both of these groups exhibited lower cfDNA levels than PE with treatment (*p* < 0.05 and *p* = 0.0146, respectively; Figure 1B). Moreover, when HDP women prescribed antihypertensive therapy were grouped into treatment-responsive versus treatment-non-responsive (see Materials and Methods section for clinical criteria used on the classification of antihypertensive therapy responsiveness), total cfDNA was elevated in both subgroups of GH and PE patients compared to HP (all *p* < 0.0001; Figure 1C); however, cfDNA levels were slightly higher in treatment-non-responsive (rTTM no) versus responsive (rTTM yes) GH (*p* = 0.0445; Figure 1C).

**Figure 2:**
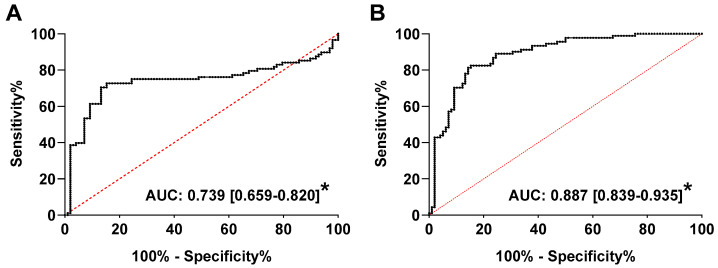
Receiver-operating characteristic (ROC) curves for total cell-free DNA (cfDNA) concentration in plasma of women with gestational hypertension (GH, **A**) or preeclampsia (PE, **B**) compared to women with healthy pregnancies (HP). Values are area under the curve (AUC) {95% confidence interval}. * *p* < 0.0001 versus HP group.

Receiver-operating characteristic (ROC) curves were then produced to determine the sensitivity and specificity of plasma total cfDNA as a diagnostic marker of GH or PE compared to HP. As depicted in Figure 2A,B, respectively, the area under the curve (AUC) for total cfDNA in PE versus HP (AUC = 0.887) was higher than in GH versus HP (AUC = 0.739) (both *p* < 0.0001). Using a cutoff value >160.0 ng/mL, the corresponding sensitivity, specificity, and likelihood ratio were 82.4% {95% CI: 73.3–88.9%}, 84.7% {95% CI: 76.3–90.5%}, and 5.4 in PE and 71.6% {95% CI: 61.4–80.0%}, 84.7% {95% CI: 76.3–90.5%}, and 4.7 in GH, respectively. However, total cfDNA did not distinguish between GH and PE (AUC: 0.576 {95% CI: 0.492–0.660}, *p* = 0.0789). As GA at enrollment/sampling, and consequently the time point of cfDNA measurement, ranged from 36 {35–37} to 37 {37–38} weeks (Table 1), we performed an additional analysis comparing total cfDNA in a concomitant diagnosis of HDP versus HP over late gestation (Supplementary Materials Figure S2). Notably, levels were higher and lower than 160 ng/mL in HDP and HP, respectively, during the entire GA interval of total cfDNA measurement (all *p* < 0.05).

**Figure 3:**
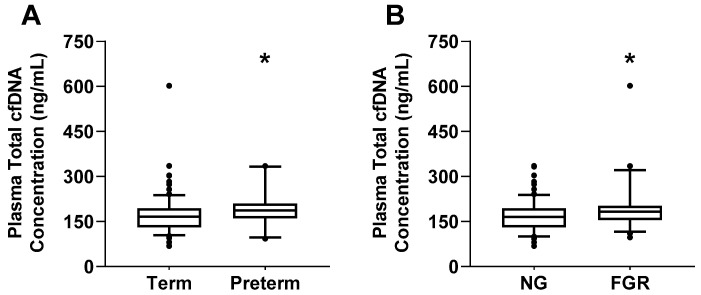
Total cell-free DNA (cfDNA) concentration in plasma of women who delivered at term (≥37 weeks of gestation) and those who delivered preterm (<37 weeks of gestation) (**A**). Total cfDNA levels in pregnant women who delivered normal growth newborns (NG, >10th percentile) and those who delivered fetal growth restricted newborns (FGR, < 10th percentile) (**B**). Box and whiskers are median {interquartile range, 5–95% percentile}. * *p* < 0.05 versus term or NG group based on Mann-Whitney test.

Next, we evaluated the univariate relationship between plasma total cfDNA concentration and clinical characteristics in all studied subjects by performing correlation analyses. As shown in Table 2, total cfDNA correlated positively with SBP, DBP, AST, and LDH, and negatively with HR, platelet count, GA at delivery, and newborn weight (all *p* < 0.05). We also observed differences in total cfDNA levels across the population cohort in women who delivered preterm (<37 weeks of gestation) compared to those who delivered at term (≥37 weeks of gestation) (Figure 3A, *p* = 0.0051). Total cfDNA levels were also elevated in pregnant women who delivered fetal growth-restricted (FGR) newborns (<10th weight corrected for GA percentile) compared to those who delivered normal growth (NG) newborns (>10th weight corrected for GA percentile; Figure 3B, *p* = 0.0093).

To evaluate the independent predictive capacity of plasma total cfDNA for the diagnosis of GH and PE, we performed multivariable logistic regression analyses. A clinically-based model (Model 1) was optimized including only clinical characteristics that would be available to the physician in a regular prenatal office visit (Supplementary Materials Table S1); whereas a mechanistic model (Model 2) included characteristics available at the end of the patient’s pregnancy course (e.g., GA at delivery and newborn weight; Supplementary Materials Table S2). In both models, a 1 ng/mL increase in plasma total cfDNA concentration was consistently associated with a 3–6% increase in the likelihood of having GH or PE independently of the model applied (Table 3).

Finally, we performed correlation analyses to evaluate the univariate relationship between plasma total cfDNA concentration and other circulating factors previously demonstrated by our research team to play a role in the pathophysiology of HDP [33,34,35,36,37,38,39,40]. The comparison of plasma levels of these biomarkers among the HP, GH, and PE subjects included in this study is depicted in Supplementary Materials Table S3. As shown in Table 4, total cfDNA correlated positively with cyclic guanosine monophosphate (cGMP), soluble fms-like tyrosine kinase-1 (sFLT-1), soluble endoglin (sEndoglin), matrix metalloproteinase (MMP)-2, tissue inhibitor of metalloproteinase (TIMP)-1, and TIMP-2 and negatively with the ratio of MMP-2/TIMP-2 (all *p* < 0.05).

## 3. Discussion

The novel findings reported here are that plasma total cfDNA levels were similarly increased in PE and GH compared to HP. Interestingly, total cfDNA levels were elevated in both GH and PE versus HP independent of fetal sex, and in more severe versus less severe HDP cases classified according to responsiveness to antihypertensive therapy. In addition, total cfDNA was independently associated with GH and PE, and a cutoff value of 160 ng/mL provided appropriate sensitivity and specificity values for detecting GH and PE compared to HP. This was accomplished by applying a simple, rapid, and sensible spectrometry-based test for quantifying total cfDNA. Moreover, high total cfDNA was significantly associated with adverse maternal-fetal outcomes (high blood pressure, low platelet count, preterm delivery, FGR) and with high circulating prohypertensive factors (sFLT-1, sEndoglin, MMP-2).

Since Lo and colleagues (1997) demonstrated the presence of fetal cfDNA in the maternal circulation [10], the investigation of cell-free nucleic acids as biomarkers for maternal-fetal diseases has been the subject of several articles. While many studies in HDP have focused on fetal cfDNA in maternal circulation, this subtype comprises only a small fraction of total cfDNA; the majority of circulating cfDNA is of maternal hematopoietic cell and adipocyte origin [41]. Moreover, nearly all of these studies on fetal cfDNA rely on the quantification of Y-chromosome specific gene sequences by quantitative real time-polymerase chain reaction (qPCR) and, thus, were only able to assess fetal cfDNA in pregnant women bearing male fetuses [42]. Furthermore, three recent systematic reviews concluded that, due to the heterogeneity of enrolled populations, lack of standardized quantification protocols, and difficulty in interpreting published results, the statistical robustness and clinical relevance of circulating cfDNA as an independent biomarker for the prediction and/or progression monitoring of PE is uncertain [43,44,45].

Therefore, we designed a study to measure total circulating levels of cfDNA in HDP as well as normal pregnancy using fluorescence spectrometry. This technique is specific to double-stranded DNA (dsDNA) fragments and strongly correlates with qPCR [46]. In addition to quantifying cfDNA in both male and female-bearing pregnancies, it has the advantages of being inexpensive and less time-consuming in terms of both performance and data analysis. We found that plasma total cfDNA concentrations were elevated in PE compared to HP. Prior studies have also described higher maternal or total cfDNA in the circulation of pregnant women with established PE [11,12,13,14,15,16,17,18,19,20,21,22,23]. Among these reports, just Rafaeli-Yehudai’s (2018) [18] and Kwak’s (2020) [14] studies have used fluorescence spectrometry to assess circulating total cfDNA in PE. However, only two studies have previously examined cfDNA in established GH [13,14], and they did not find differences in circulating total or fetal cfDNA assessed by qPCR. In contrast, here we showed for the first time elevated total cfDNA levels in GH compared to HP when directly quantified via fluorescence spectrometry. Additionally, to our knowledge, we are the first to demonstrate that total cfDNA is higher in both GH and PE independently of fetal sex in the same population cohort, although it has been shown that circulating fetal cfDNA is increased in PE versus normal pregnant women carrying either a male [11,15,22,23,24,27,47,48,49,50,51] or female [52] fetus in distinct populations by applying sex-dependent gene sequences in qPCR.

Despite that many maternal circulating biomarkers have been suggested for identifying pregnancies at risk of HDP [6,7], most are either not discriminative enough or use quantification methods that are too demanding to be used clinically. Here we found that a cutoff value of 160 ng/mL for plasma total cfDNA concentration provides good sensitivity and specificity (70–85%) for a concomitant diagnosis of HDP, with slightly stronger fidelity for PE compared to GH. Even when adjusted for either clinical or mechanistic variables, total cfDNA remained independently associated with both GH and PE: in this analysis, every 1 ng/mL increase in cfDNA was associated with a ~4% increase in the probability of diagnosing either HDP. This observation is in line with prior reports showing that cfDNA levels become elevated before the appearance of the clinical symptoms of PE [19,53,54,55,56], although some studies could not replicate these findings [57,58,59,60,61]. These divergent results may be related to differences in specific gene selection for qPCR used in these previous studies. The use of bulk DNA measurement by fluorescence spectrometry described here may mitigate this heterogeneity, and the relatively low cost and ease of performance make it more appealing for clinical use. Collectively, these findings suggest that cfDNA may have utility for detecting the concomitant diagnosis of HDP.

Earlier reports also indicate that total cfDNA levels are positively correlated with disease severity in PE [11,13,17,22,23]. While plasma total cfDNA concentration did not appear to have a stepwise increase among HP, GH, and PE groups, our study is the first to assess the impact of antihypertensive therapy on cfDNA levels in HDP. Patients not requiring antihypertensive treatment for blood pressure control and those taking antihypertensive drugs but considered responsive to the therapy regiment frequently present less severe disease compared to those considered non-responsive to the antihypertensive therapy regiment. Accordingly, we found that total cfDNA was increased in GH requiring antihypertensive therapy compared to HP and GH not requiring antihypertensive therapy and was elevated in PE requiring antihypertensive treatment compared to both of these groups. In addition, GH women non-responsive to the antihypertensive therapy exhibited higher total cfDNA levels than their non-responsive counterparts. Furthermore, increased total cfDNA was significantly associated with increased SPB, DBP, AST, and LDH, and with decreased blood platelet count, GA at delivery, and newborn weight. Likewise, we noted that total cfDNA levels were elevated in pregnant women who delivered preterm and FGR newborns. Equivalent associations have been described in relation to blood pressure, enzymes linked to tissue damage, platelet count, GA at delivery, newborn weight, preterm delivery, and FGR [11,12,14,15,16,17,57,62,63]. Surprisingly, increased total cfDNA was also associated with decreased HR. However, this may reflect the effects of antihypertensive drugs, specially methyldopa [64,65], on reducing heart rate, because when we excluded the HDP patients under antihypertensive therapy from the correlation analysis, the association between total cfDNA and HR was no longer statistically significant (Rho = −0.1419, 95% CI {−0.320 to 0.046}, *p* = 0.1375). Taken together, these findings suggest that cfDNA may serve as a prognostic marker of clinical severity and maternal-fetal outcomes in HDP.

As a prospective observational study, whether cfDNA plays a causal role in HDP cannot be determined; however, several mechanistic insights are evident that may shape upcoming studies addressing this limitation. In particular, increased total cfDNA correlated with increased MMP-2, TIMP-2, TIMP-1, sFLT-1, sEndoglin, and cGMP, as well as with a decreased ratio of MMP-2/TIMP-2. Similar associations have been reported concerning sFlt-1 and sEndoglin in PE [11,17]. Whereas elevated MMP-TIMP levels have been linked to hypertension by inducing the vasoconstrictor arm of the endothelin system, elevated sFLT-1 and sEndoglin levels may affect vascular function by acting on the nitric oxide-cGMP signaling pathway in PE [8,66]. As shedding of the placental and endothelium glycocalyx degraded by MMP-TIMP overactivity may result in increases in circulating cfDNA and sFLT-1, future studies will evaluate this potential mechanism by measuring glycocalyx components such as heparan sulfate, hyaluronic acid, and syndecans [67,68]. Furthermore, activation of inflammatory cells and platelets as part of a sterile inflammatory process underlying HDP may result in the release of cfDNA by neutrophils and/or platelets, which may lead to subsequent high risk for thrombotic complications [31,69]. Future studies will also require analysis of markers of neutrophil extracellular trap formation and platelet activation and function in order to understand the origin and specific nature of increased cfDNA release in the circulation of HDP women.

Several other limitations are important to acknowledge in the interpretation of our results. In addition to not identifying the source of cfDNA and determining whether the extracted cfDNA actually elicits endothelial and/or vascular dysfunction in isolated blood vessels, total cfDNA correlated with blood pressure but not with plasma creatinine or proteinuria, indicating that increased total cfDNA in HDP may relate to mechanisms leading to hypertension and not renal damage. Indeed, ROC curves plotted with plasma total cfDNA levels in PE patients against those in GH patients demonstrated that this marker is not able to distinguish between PE and GH, suggesting that total cfDNA has value for diagnosing new-onset hypertension during pregnancy. Moreover, because PE may develop as soon as 20 weeks of gestation and rapidly progress, PE patients are frequently referred to tertiary hospitals for follow-up and possibly delivery at earlier GAs than GH and HP. Thus, the lower GA at enrollment in PE versus GH and HP reported here is most likely due to early referral from healthcare providers in the metropolitan area of Ribeirao Preto to our academic medical institution. Nonetheless, total cfDNA was elevated in a concomitant diagnosis of HDP compared to HP during the entire interval concerning the GA at enrollment/sample collection period. Prior studies measuring maternal or total cfDNA at different GAs diverge on whether levels increase or remain steady across gestation in HP or PE depending on the selected gene for qPCR [14,19,20,54,60,62]. Future studies should prospectively assess fluorescence spectrometry-based cfDNA levels during pregnancy to rigorously evaluate its diagnostic and prognostic values.

As aforementioned, the methodology used for the measurement of cfDNA significantly affects the obtained results. As cfDNA is generated during the clotting process, circulating serum cfDNA levels may be confounded by cfDNA produced ex vivo [70,71] compared to levels detected in plasma. Since patients with PE often have disturbances in the coagulation process [72], plasma should be the tissue of choice to avoid misinterpretations of study data. Finally, divergent findings among studies can also be explained by the sample sizes, population demographics, GA at sampling, and the criteria utilized for diagnosing HDP and classifying patients into severity groups. While recently published articles may have adopted the American College of Obstetricians and Gynecologists Task Force on Hypertension in Pregnancy’s recommendations released in 2013 [73], older studies have not employed this updated guideline. Indeed, HDP were defined in our study according to the National High Blood Pressure Education Program Working Group on High Blood Pressure in Pregnancy’s recommendations released in 2000 [74], as enrollment was conducted between 2006 and 2008. Therefore, our findings require confirmation in different populations.

In conclusion, we showed that circulating cfDNA is elevated in pregnant women with HDP (including GH and PE) compared to HP. In addition to proposing a cut-off value of plasma total cfDNA concentration as a sensitive and specific tool for the diagnosis of hypertension in both male and female-bearing pregnancies, we demonstrated that total cfDNA levels might vary in HDP depending on disease severity. Our results suggest that total cfDNA measurement in plasma by fluorescence spectrometry as part of routine prenatal care should be evaluated prospectively as a potential diagnostic and prognostic marker for maternal-fetal outcomes in HDP. These data also suggest that cfDNA may be involved in vascular dysfunction, exaggerated inflammatory response, and susceptibility to thrombotic events associated with HDP. These findings represent a step forward towards the establishment of plasma total cfDNA as a biomarker and pathophysiologically relevant mediator of HDP.

## 4. Materials and Methods

### 4.1. Subjects

Approval for the use of human subjects was obtained from the Institutional Review Board of the General Hospital (HC) of the Ribeirao Preto Medical School (FMRP), University of Sao Paulo (reference 4682/2006, approved date 20 June 2006). Procedures followed the institutional guidelines for designing, conducting, recording, and reporting studies involving human subjects. Enrolled human subjects voluntarily agreed to participate in this research and provided written informed consent.

Pregnant women were recruited and consecutively followed by healthcare providers in the Department of Gynecology and Obstetrics of the tertiary referral hospital HC-FMRP from September 2006 to November 2008. Two hundred seventy seven pregnant women were included in this study: 98 with uncomplicated HP, 88 with GH, and 91 with PE. HDP were diagnosed in accordance with the guidelines of the National High Blood Pressure Education Program Working Group on High Blood Pressure in Pregnancy [74]. Gestational hypertension was defined as pregnancy-induced hypertension (>140 mmHg systolic or >90 mmHg diastolic on more than 2 measurements taken at least 6 h apart) without proteinuria in a woman after 20 weeks of gestation, with blood pressure levels returning to normal by 12 weeks postpartum. Preeclampsia was defined as increased blood pressure with significant proteinuria (>0.3 g/24 h) in a woman after 20 weeks of gestation. Babies born before 37 weeks of gestation were considered preterm, and those with birth weight <10th percentile were categorized as FGR. No women with chronic hypertension, with or without superimposed PE, were included in this study. Exclusion criteria also included twin or multiple pregnancies, any evidence of previous or ongoing medical illness, and maternal death.

Demographic and clinical information, including results of routine laboratory tests, of pregnant women and respective newborns were collected from medical records.

### 4.2. Disease Severity Criteria

We used antihypertensive therapy as a proxy of disease severity. Patients with HDP were first categorized according to the need for drug therapy for blood pressure control. Patients presenting SBP between 140 and 145 mmHg and DBP between 90 and 95 mmHg, as assessed by blood pressure curve, did not receive antihypertensive therapy because it may reduce not only maternal blood pressure, but also utero-placental blood flow, potentially leading to unfavorable fetal outcomes. However, antihypertensive therapy was prescribed to patients when they exhibited SBP higher than 145 mmHg and/or DBP higher than 95 mmHg. Next, those patients with HDP requiring antihypertensive therapy were further classified as responsive or non-responsive to a standardized therapy regimen as follows: initial treatment with methyldopa (1000–1500 mg per day); addition or substitution of nifedipine (40–60 mg per day) for lack of blood pressure response to methyldopa; rescue use of hydralazine (5–30 mg) in cases of acute hypertensive syndromes. All patients in this study were monitored at least weekly for signs and symptoms of HDP. The presence of at least one of the following criteria was considered to classify a patient as non-responsive to the antihypertensive therapy:

- Clinical symptoms including blurred vision, persistent headache or scotomata, persistent right upper quadrant or epigastric pain;
- SBP >140 mmHg and DBP > 90 mmHg;
- Abnormal results in clinical laboratory tests such as those present in HELLP syndrome; or proteinuria > 2.0 g per 24 h; creatinine > 1.2 mg per 100 mL; blood urea nitrogen > 30 mg per 100 mL; aspartate aminotransferase > 70 U/L; and alanine aminotransferase > 60 U/L;
- Fetal hypoactivity or nonreactive fetus, as revealed by cardiotocography; intrauterine growth restriction, oligoamnio, abnormal biophysical profile score, and Doppler velocimetry abnormalities, as evaluated by ultrasound.

### 4.3. Sample Collection

Maternal venous blood was sampled at study enrollment during a prenatal office visit conducted after the 20 weeks of gestation. Blood was collected into BD Vacutainer^®^ tubes containing ethylenediaminetetraacetic acid (EDTA) as an anticoagulant (Becton-Dickinson, Sao Paulo, SP, Brazil). These tubes were immediately centrifuged at 2000 g for 10 min at room temperature, and plasma samples were stored at −70 °C until used to measure circulating total cfDNA concentrations. Circulating factors including adiponectin, asymmetric dimethylarginine (ADMA), cGMP, leptin, malondialdehyde–thiobarbituric acid reactive substances (MDA-TBARS), MMP-2, MMP-9, myeloperoxidase, nitrite, sEndoglin, sFLT-1, TIMP-1, and TIMP-2 were quantified as previously described [33,34,35,36,37,38,39,40].

### 4.4. Extraction and Quantification of DNA

Total cfDNA was extracted from 200 μL of plasma samples using a QIAamp DNA blood mini kit (Qiagen, Hilden, NW, Germany) according to the manufacturer. Quantification of total cfDNA was carried out in triplicate using Quant-iT™ PicoGreen^®^ dsDNA detection kit (Molecular Probes, Eugene, OR, USA) according to the manufacturer’s instructions. Briefly, standards (DNA concentration in standard curve ranged from 0 to 1000 ng/mL) and samples (DNA in final eluted product from extraction protocol) were mixed with the PicoGreen reagent (1:1), and the fluorescence intensity was measured on a spectrofluorometer (SpectraMax^®^ Gemini™ EM, Molecular Devices, Sunnyvale, CA, USA) at an emission wavelength of 520 nm and excitation of 480 nm [75].

### 4.5. Statistical Analysis

Data distribution was analyzed by D’Agostino-Pearson omnibus normality test. Categorical variables were compared by χ^2^ test. Continuous variables were compared by Mann-Whitney, one-way analysis of variance, or Kruskal-Wallis test followed by a multiple comparisons test as appropriate. ROC curves were constructed with the calculation of AUC, sensitivity, specificity, and likelihood ratio at different cutoff levels. The relationships between plasma total cfDNA levels and clinical or biochemical characteristics were analyzed using Spearman correlation. Figures and statistical analysis were prepared with GraphPad Prism version 8.4.3 (GraphPad Software, San Diego, CA, USA). Multivariable logistic regression models were constructed using Stata version 16 (StataCorp LLC, College Station, TX, USA) with a priori inclusion of predictors to produce a clinically-based model (drawn from only parameters that would be available to a physician at the time of a prenatal office visit) as well as a mechanistic model (drawn from all clinical data available at the end of the patient’s pregnancy course, including data regarding delivery). The non-linearity of gestational age was modeled using linear splines, with <10% difference in the coefficient for cfDNA (data not shown). Goodness of fit was assessed by Hosmer-Lemeshow testing. Model prediction was assessed based on the area under the ROC curve. A *p* value of <0.05 was considered statistically significant.

## Figures and Tables

**Table 1:** Demographic and clinical characteristics of study subjects.

Parameter	Healthy Pregnancy (*n* = 98)	Gestational Hypertension (*n* = 88)	Preeclampsia (*n* = 91)
At enrollment			
Age (years)	26.2 ± 0.7	26.6 ± 0.7	27.2 ± 0.8
Race (white)	62 (63.3)	62 (70.5)	62 (68.1)
Current smoking	10 (10.2)	11 (12.5)	8 (8.8)
Body mass index (kg/m^2^)	29.7 {28.1–30.5}	33.3 {31.6–35.1} *	32.1 {30.1–33.8} *
Primiparity	34 (34.7)	36 (40.9)	37 (40.7)
Gestational age (weeks)	37 {37–38}	37 {37–38}	36 {35–37} *^,&^
During follow-up			
Systolic blood pressure (mm Hg)	113.3 {110.0–115.0}	131.0 {130.0–135.0} *	140.0 {136.7–144.0} *^,&^
Diastolic blood pressure (mm Hg)	72.4 ± 0.9	84.8 ± 1.3 *	88.9 ± 1.1 *^,&^
Antihypertensive therapy			
Under treatment	0 (0)	67 (76.1)	78 (85.7)
Non-responsiveness	N/A	13 (14.8)	39 (42.9) ^&^
Heart rate (bpm)	80.7 {80.0–82.0}	82.0 {80.0–83.3}	81.0 {80.0–82.7}
Creatinine (µM)	N/D	53.0 {53.0–53.0}	61.9 {53.0–61.9} ^&^
Proteinuria (mg/24 h)	N/D	149.5 {121.0–169.6}	449.9 {346.5–775.9} ^&^
Hematocrit (%)	35.9 {33.7–37.0}	36.8 {35.7–37.0}	36.9 {35.1–37.5}
Hemoglobin (g/dL)	11.9 {11.1–12.4}	12.1 {11.9–12.4}	12.1 {11.9–12.4}
White blood cells (×10^3^/mm^3^)	11.4 {9.7–12.7}	9.9 {9.4–10.5}	9.9 {8.9–10.6}
Neutrophils (% of WBC)	69.0 {65.0–73.5}	70.3 {66.4–72.0}	71.0 {68.0–73.0}
Platelets (×10^3^/mm^3^)	228.4 ± 14.7	223.6 ± 58.2	211.2 ± 67.2
Fasting glucose (g/dL)	69.5 {68.0–76.0}	79.3 {76.0–82.0} *	77.0 {73.5–80.0} *
Alanine transaminase (U/L)	N/D	14.0 {11.0–15.0}	14.0 {12.0–15.0}
Aspartate aminotransferase (U/L)	N/D	17.0 {15.0–18.0}	17.0 {16.0–19.0}
Lactate dehydrogenase (U/L)	N/D	436.0 {364.0–484.0}	429.5 {389.0–466.0}
At delivery			
Gestational age (weeks)	39 {39–39}	39 {38–40}	37 {37–38} *^,&^
Preterm birth	1 (1.0)	6 (6.8)	31 (34.1) *^,&^
Newborn weight (g)	3380 {3125–3465}	3160 {3020–3290}	2900 {2620–3050} *^,&^
Fetal growth restriction	7 (7.1)	18 (20.5) *	30 (33.0) *
Placental weight (g)	562.5 ± 14.7	592.3 ± 13.1	543.0 ± 17.5

N/A, not applicable; N/D, not determined (however, negative urine dipstick test). Values are mean ± standard error of the mean for parametric variables; median {95% confidence interval} for non-parametric variables; and frequency (percentage) for categorical variables. * *p* < 0.05 versus healthy pregnancy and ^&^ *p* < 0.05 versus gestational hypertension based on Mann-Whitney, ordinary one-way analysis of variance, Kruskal-Wallis, or χ2 test, as appropriate, followed by correction for multiple comparisons.

**Table 2:** Relationships between total cfDNA and clinical characteristics in all studied subjects.

Parameter	Rho	95% CI	*p*
Age (years)	0.043	−0.086 to 0.170	0.5032
Systolic blood pressure (mm Hg)	0.420	0.311 to 0.519	<0.0001
Diastolic blood pressure (mm Hg)	0.374	0.260 to 0.478	<0.0001
Heart rate (bpm)	−0.144	−0.265 to −0.018	0.0213
Creatinine (μM)	0.031	−0.122 to 0.182	0.6843
Proteinuria (mg/24 h)	0.093	−0.111 to 0.290	0.3568
Hematocrit (%)	0.061	−0.074 to 0.194	0.3595
Hemoglobin (g/dL)	0.056	−0.080 to 0.189	0.4083
White blood cells (×10^3^/mm^3^)	−0.041	−0.185 to 0.104	0.5672
Neutrophils (% of WBC)	0.079	−0.091 to 0.244	0.3498
Platelets (×10^3^/mm^3^)	−0.172	−0.310 to 0.027	0.0167
Fasting glucose (g/dL)	0.103	−0.044 to 0.245	0.1577
Alanine transaminase (U/L)	0.154	−0.047 to 0.342	0.1213
Aspartate aminotransferase (U/L)	0.188	0.033 to 0.333.	0.0146
Lactate dehydrogenase (U/L)	0.334	0.028 to 0.582	0.0288
Gestational age at enrollment (weeks)	−0.046	−0.169 to 0.077	0.4493
Body mass index at enrollment (kg/m^2^)	0.092	−0.035 to 0.216	0.1443
Gestational age at delivery (weeks)	−0.181	−0.307 to −0.049	0.0058
Newborn weight (g)	−0.256	−0.375 to −0.129	<0.0001
Placental weight (g)	−0.039	−0.174 to 0.098	0.5699

Values are the Spearman’s Rho, 95% confidence interval (CI), and *p* values for the correlation between plasma total cell-free DNA concentration (cfDNA, ng/mL) and the respective clinical parameter.

**Table 3:** Multivariable logistic regression analysis for plasma total cfDNA.

	Model 1		Model 2	
	Gestational Hypertension	Preeclampsia	Gestational Hypertension	Preeclampsia
Odds ratio	1.02	1.05	1.02	1.10
95% Confidence interval	1.01–1.03	1.03–1.07	1.01–1.04	1.04–1.16
*p*	<0.001	<0.001	0.007	0.001

Model 1 adjusted for body mass index and gestational age at enrollment, with area under the curve (AUC) > 0.810. Model 2 adjusted for systolic blood pressure, body mass index, gestational age at delivery, and newborn weight, with AUC > 0.950.

**Table 4:** Relationships between total cfDNA and circulating factors in all studied subjects.

Plasma Parameter	Rho	95% CI	*p*
Nitrite (nM)	−0.136	−0.277 to 0.011	0.0612
ADMA (µM)	−0.037	−0.264 to 0.193	0.7470
cGMP (nM)	0.239	0.027 to 0.430	0.0234
sFLT−1 (ng/mL)	0.299	0.094 to 0.479	0.0038
sEndoglin (ng/mL)	0.296	0.097 to 0.473	0.0032
Myeloperoxidase (ng/mL)	0.003	−0.125 to 0.132	0.9588
MDA-TBARS (nM)	−0.019	−0.169 to 0.131	0.7975
Leptin (ng/mL)	0.082	−0.093 to 0.252	0.3433
Adiponectin (µg/mL)	0.066	−0.109 to 0.237	0.4454
MMP−2 (ng/mL)	0.228	0.109 to 0.341	0.0001
TIMP-2 (ng/mL)	0.286	0.170 to 0.394	<0.0001
MMP-2/TIMP-2 ratio	−0.160	−0.277 to −0.038	0.0082
MMP-9 (ng/mL)	0.114	−0.008 to 0.233	0.0596
TIMP-1 (ng/mL)	0.278	0.161 to 0.386	<0.0001
MMP-9/TIMP-1 ratio	0.042	−0.080 to 0.163	0.4869

ADMA, asymmetric dimethylarginine; cGMP, cyclic guanosine monophosphate; MDA-TBARS, malondialdehyde–thiobarbituric acid reactive substances; MMP-2, matrix metalloproteinase-2; MMP-9, matrix metalloproteinase-9; sEndoglin, soluble endoglin; sFLT-1, soluble fms-like tyrosine kinase-1; TIMP-1, tissue inhibitor of metalloproteinase-1; TIMP-2, tissue inhibitor of metalloproteinase-2. Values are the Spearman’s Rho, 95% confidence interval (CI), and *p* values for the correlation between plasma total cell-free DNA concentration (cfDNA, ng/mL) and the respective circulating factor.

## Data Availability

The authors confirm that data supporting the findings of this study are presented within the article and the Supplement Materials.

## Supplementary Materials

Supplementary materials can be found at https://www.mdpi.com/1422-0067/22/2/564/s1.

## Abbreviations

ADMA	Asymmetric dimethylarginine
ALT	Alanine transaminase
AST	Aspartate aminotransferase
AUC	Area under the curve
BMI	Body mass index
cfDNA	Cell-free deoxyribonucleic acid
cGMP	Cyclic guanosine monophosphate
CI	Confidence interval
DBP	Diastolic blood pressure
dsDNA	Double-stranded deoxyribonucleic acid
FGR	Fetal growth restriction
GA	Gestational age
GH	Gestational hypertension
HC-FMRP	General Hospital of the Ribeirao Preto Medical School
HDP	Hypertensive disorders of pregnancy
HELLP	Hemolysis, elevated liver enzymes and low platelet count syndrome;
HP	Healthy pregnancy
HR	Heart rate
LDH	Lactate dehydrogenase
MDA-TBARS	Malondialdehyde–thiobarbituric acid reactive substances
MMP	Matrix metalloproteinase
NG	Normal growth
OR	Odds ratio
PE	Preeclampsia
qPCR	Quantitative real time-polymerase chain reaction
ROC	Receiver-operating characteristic
rTTM	Responsive to treatment
SBP	Systolic blood pressure
sEndoglin	Soluble endoglin
sFLT	Soluble fms-like tyrosine kinase
TIMP	Tissue inhibitor of metalloproteinase
TTM	Treatment
