# Supplementary material for: Circulating Total Cell-Free DNA Levels Are Increased in Hypertensive Disorders of Pregnancy and Associated with Prohypertensive Factors and Adverse Clinical Outcomes
Source: Int J Mol Sci. 2021 Jan 8;22(2):564. doi: 10.3390/ijms22020564 (PMC7826953; doi:10.3390/ijms22020564)
Supplement: Supplementary file 1 [file ijms-22-00564-s001.pdf]

SUPPLEMENTARY MATERIALS

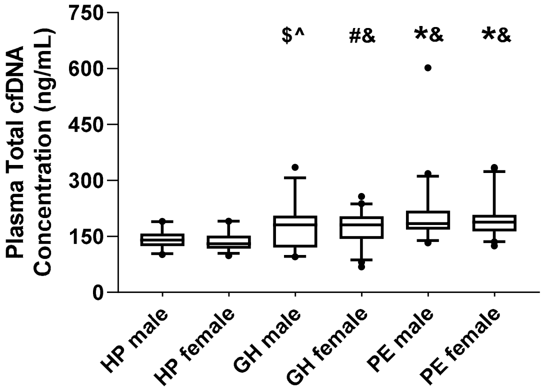

Figure S1. Total cell-free DNA (cfDNA) concentration in plasma of women with healthy pregnancies (HP), gestational hypertension (GH), or preeclampsia (PE) according to male and female fetal sex. From the 277 subjects included in this study, 61 HP (44.3% males), 85 GH (42.4% males), and 90 PE (53.3% males) women had fetal sex reported after delivery in their medical reports (P>0.05). Box and whiskers are median {interquartile range, 5-95% percentile}. $P<0.05 versus HP male group, ^P<0.01 versus HP female group, #P<0.01 versus HP male, *P<0.0001 versus HP male group, &P<0.0001 versus HP female group based on Kruskal-Wallis test with Dunn's multiple comparisons correction.

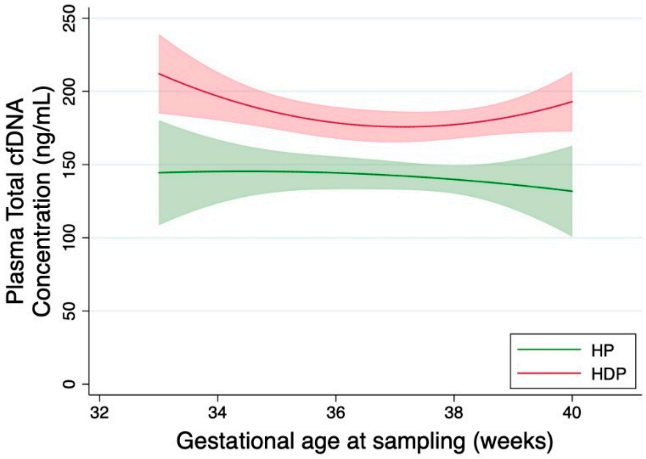

Figure S2. Total cell-free DNA (cfDNA) concentration in plasma of women with hypertensive disorders of pregnancy (HDP, comprising the diagnosis of gestational hypertension or preeclampsia) versus healthy pregnancies (HP) over gestational age at enrollment/blood collection. Lines are mean values and range {95% confidence interval}.

Table S1. Multi-variable logistic regression analysis for model 1.

	Gestational Hypertension			Preeclampsia		
	**OR**	**95% CI**	**P**	**OR**	**95% CI**	**P**
Total cfDNA (ng/mL)	1.02	1.01-1.03	<0.001	1.05	1.03-1.07	<0.001
Body Mass Index (kg/m^2)	1.21	1.11-1.32	<0.001	1.16	1.06-1.27	0.001
Gestational Age at enrollment (weeks)	0.84	0.73-0.98	0.03	0.74	0.60-0.90	0.002

For the outcome of gestational hypertension, the model had area under the curve (AUC)=0.816 and Hosmer-Lemeshow goodness-of-fit statistic=0.475. For the outcome of preeclampsia, the model had AUC=0.911 and goodness-of-fit statistic=0.001. Modeling of gestational age as a three-knot linear spline improved AUC (to 0.940) and goodness-of-fit (to 0.065) with no substantive change in the coefficient (OR=1.06) or P-value (P<0.001) for total cfDNA. OR, odds ratio; CI, confidence interval.

Table S2. Multi-variable logistic regression analysis for model 2.

	Gestational Hypertension			Preeclampsia		
	**OR**	**95% CI**	**P**	**OR**	**95% CI**	**P**
Total cfDNA (ng/mL)	1.02	1.01-1.04	0.007	1.10	1.04-1.16	0.001
Systolic Blood Pressure (mmHg)	1.20	1.11-1.30	<0.001	1.31	1.13-1.52	<0.001
Body Mass Index (kg/m^2)	1.19	1.06-1.35	0.004	1.45	1.13-1.86	0.004
Gestational Age at delivery (weeks)	0.98	0.63-1.52	0.923	0.76	0.33-1.72	0.505
Newborn Weight (g)	1.00	1.00-1.00	0.712	1.00	1.00-1.00	0.892

For the outcome of gestational hypertension, the model had area under the curve (AUC)=0.950 and Hosmer-Lemeshow goodness-of-fit statistic=0.237. For the outcome of preeclampsia, the model had AUC=0.992 and goodness-of-fit statistic=1.000. OR, odds ration; CI, confidence interval.

Table S3. Circulating factors in study subjects.

Plasma Parameter	Healthy Pregnancy	Gestational Hypertension	Preeclampsia
Nitrite (nM)	140.50 {98.16-173.50]	76.22 {66.27-122.50] *	85.20 {66.42-98.73] *
ADMA (μM)	2.13 {2.05-2.20]	2.13 {2.08-2.19]	2.17 {2.10-2.21]
cGMP (nM)	11.94 {9.75-14.00]	16.97 {13.87-21.88] *	14.83 {12.54-17.77] *
sFLT-1 (ng/mL)	3.33 {2.19-4.29]	5.83 {3.96-8.12] *	11.31 {6.05-14.89] *
sEndoglin (ng/mL)	22.65 {17.09-27.55]	24.48 {21.12-27.10]	34.13 {27.83-43.21] *&
Myeloperoxidase (ng/mL)	2.85 {2.45-3.54]	2.37 {2.21-3.00]	2.58 {2.17-3.12]
MDA-TBARS (nM)	5.09 {4.55-5.43]	5.15 {4.42-5.66]	4.92 {4.50-5.32]
Leptin (ng/mL)	22.54 {19.07-26.80]	17.86 {13.80-22.32]	29.65 {16.80-36.87]
Adiponectin (μg/mL)	17.53 {14.50-18.84]	17.79 {16.01-21.01]	18.83 {15.94-23.59]
MMP-2 (ng/mL)	238.10 {227.90-247.70]	260.30 {237.80-269.40] *	268.70 {255.40-281.20] *
TIMP-2 (ng/mL)	156.90 {144.80-165.60]	157.00 {146.50-167.80]	183.00 {167.30-190.60] *&
MMP-2/TIMP-2 ratio	1.54 {1.47-1.58]	1.63 {1.55-1.67] *	1.48 {1.44-1.53] &
MMP-9 (ng/mL)	198.60 {161.40-244.40]	216.60 {175.80-264.40]	216.40 {161.60-276.80]
TIMP-1 (ng/mL)	138.90 {133.30-149.00]	154.40 {147.20-164.10] *	178.80 {168.40-186.70] *&
MMP-9/TIMP-1 ratio	1.42 {1.22-1.91]	1.39 {1.17-1.65]	1.23 {0.92-1.51]

ADMA, asymmetric dimethylarginine; cGMP, cyclic guanosine monophosphate; MDA-TBARS, malondialdehyde–thiobarbituric acid reactive substances; MMP-2, matrix metalloproteinase-2; MMP-9, matrix metalloproteinase-9; sEndoglin, soluble endoglin; sFLT-1, soluble fms-like tyrosine kinase-1; TIMP-1,

tissue inhibitor of metalloproteinase-1; TIMP-2, tissue inhibitor of metalloproteinase-2. Values are median {95% confidence interval}. *P<0.05 versus healthy pregnancy and $^{\&}$P<0.05 versus gestational hypertension based on Kruskal-Wallis test followed by Dunn's multiple comparisons correction.
